# MKP‐2 Genetic Depletion Modulates Adipose Lipid Composition, Cellular Senescence, and Aging in Mice

**DOI:** 10.1111/acel.70794

**Published:** 2026-11-03

**Authors:** Morgan Welch, Nabin Ghimire, Shauri Smith, Cassandra Secunda, Alexis Fink, Alissar Sleiman, Laura Chambliss, Christopher Pinckard, Chloe Reyes, Priya Katta, Anna Ai, Benjamin R. Harrison, Ahmed Lawan

**Affiliations:** ^1^ Department of Biological Sciences University of Alabama in Huntsville Huntsville Alabama USA; ^2^ Department of Anesthesiology and Pain Medicine University of Washington Seattle Washington USA

**Keywords:** adipose tissue, aging, MAPK, MKP‐2, senescence

## Abstract

Aging is characterized by a gradual decline in physiological functions, impaired cellular repair mechanisms, and increased disease susceptibility. Mitogen‐activated protein kinase phosphatase‐2 (MKP‐2) regulates diverse biological functions, including regulation of infection, brain activity, nutrient metabolism, and cell‐cycle progression, but its role in senescence and adipose lipid metabolism in vivo remains unclear. Here, we investigated the effects of MKP‐2 deficiency on cellular senescence and adipose lipid composition in aged mice using targeted lipidomics. MKP‐2‐deficient mouse embryonic fibroblasts (MEFs) and epididymal white adipose tissue (EWAT) exhibited reduced premature senescence. Lipidomic analysis revealed increased hexosylceramides (HexCERs) and phosphatidylglycerols (PG), and decreased diacylglycerols (DGs) in adipose tissue. These lipid alterations were accompanied by preserved lamin B1, reduced p53 phosphorylation, p16, and PAI‐1, and increased JNK and Akt phosphorylation in EWAT. MKP‐2 deficiency was also associated with extended lifespan and enhanced insulin responsiveness. Together, these studies identify MKP‐2 as a potential regulator linking adipose lipid metabolism, stress‐responsive signaling, cellular senescence, and aging, with its loss promoting a metabolically resilient adipose state that may contribute to improved systemic health and lifespan.

## Graphical abstract

Excess white adipose tissue (WAT) accumulation promotes aging by increasing the risk of insulin resistance and chronic diseases and reduced lifespan. MKP‐2 deficiency protects against premature senescence in cells and WAT and is associated with adipose lipid remodeling, improved metabolic health, and longer lifespan.

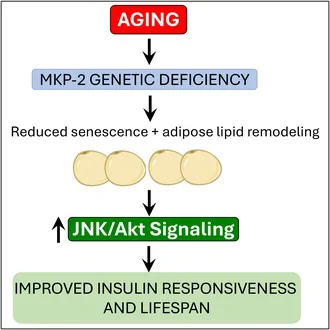

## 1 Introduction

Cells undergo many stages between proliferation, differentiation, and cell death. One such stage is known as senescence, which is a state of permanent cell cycle arrest after the cell has reached a certain age or has encountered a stress factor (Biran et al. 2017). Senescence stops the cell cycle from occurring and stops cellular proliferation. It also regulates the differentiation of cells from fibroblasts and stops irregular proliferation that may occur in tumorous cells (Gorgoulis et al. 2019; de Magalhães and Passos 2018). There are multiple forms of senescence that can occur in mammals, including oncogenic‐induced senescence, stress‐induced senescence, and replicative senescence (Regulski 2017). The senescence‐associated secretory phenotype (SASP) is characterized by altered cell size, the secretion of growth factors, cytokines, chemokines, and proteases, as well as distension of satellites, senescence‐associated heterochromatin formation, and the upregulation of some microRNAs (Calcinotto et al. 2019). Senescence is often triggered by DNA damage and telomere shortening (Chen, Chuang, and Tan 2019). DNA damage can occur through many pathways, but most leading to DNA damage occurs from stress to the cells, such as oxidative stress, radiation, and metabolic stress. Complications of metabolic stress have been linked to premature senescence in cells (Chen, Chuang, and Tan 2019). DNA damage is common in most types of senescence, especially oncogenic and stress‐induced senescence. Oncogene activation can lead to proliferative stress, which can limit tumor progression by inhibiting cell growth using the SASP as a form of paracrine signaling. Senescence can slow tumor progression by recruiting neighboring non‐senescent cells and inducing senescence, which can also affect immune cells within the tumor (Calcinotto et al. 2019). Stress‐induced senescence is induced in mid‐range levels of stress. Low levels of stress can cause damage that is easily repaired by cells, and high levels of stress often cause damage that leads to apoptosis. Stress‐induced senescence therefore stops the proliferation of damaged cells and prevents the damage from being passed onto future cell generations (Mohamad Kamal et al. 2020).

Numerous pathological conditions, such as age‐related diseases including cancer (Tabibzadeh 2021; Chew et al. 2019), Alzheimer's disease (Dineley et al. 2001; Echeverria et al. 2004), and metabolic diseases (Green et al. 2021), as well as normal aging, are linked to a loss of MAPK regulation (Freund et al. 2011). The Ras protein causes premature senescence by activating the ERK pathway (Wang et al. 2002). JNK‐deficient mouse embryonic fibroblasts (MEFs) display p53‐dependent senescence (Das et al. 2007). The MAPKs are inactivated by dephosphorylation through the actions of the dual‐specificity phosphatases (DUSPs) or MAPK phosphatases (MKPs). However, we know little about the negative regulators of MAPKs in senescence and aging. DUSP16 knockdown in liver cancer cells promotes cellular senescence through p53 and Rb activation (Zhang et al. 2015). Also, DUSP5 deletion stimulates BRAF^V600E^ oncogenic‐induced senescence in MEFs (Kidger et al. 2017). Numerous biological processes, such as the control of infection, brain activity, nutrient metabolism, and cell‐cycle progression, have been linked to the function of the Type 1 dual specificity phosphatase MKP‐2 (Lawan et al. 2012). However, the exact role of MKP‐2 function in senescence in vivo is largely unknown.

Although the precise underlying mechanisms are largely unknown, there is mounting evidence that lipid metabolism plays a critical role in preserving cell homeostasis and cell differentiation (Zeng et al. 2024; Hamsanathan and Gurkar 2022). According to recent research, senescence and aging are significantly influenced by the regulation of certain lipid species (Papsdorf and Brunet 2019). For instance, ceramides regulate cell proliferation and apoptosis caused by senescence (Zeng et al. 2024). Furthermore, certain lipid species have a role in the low‐grade inflammation linked to SASP (Palmer et al. 2019). Nevertheless, it is still unclear how important regulators of cellular senescence relate to lipid alterations linked to senescence.

According to the SASP, senescent cells interact with nearby cells to affect their microenvironment and propagate the stress response (Palmer et al. 2019). Among the many different roles of adipose tissue are energy balance, communication, endocrine function, and lipid storage (Chaurasia et al. 2016). The endocrine function of adipose tissue is impacted by cellular senescence and other aging‐related processes (Chaurasia et al. 2019). However, little is known about the processes and pathways that control adipose tissue senescence as well as SASP. Aging is frequently linked to dysfunctional adipose tissue, which is defined by decreased adipogenesis and elevated cellular senescence and inflammation (Akerblad et al. 2002; Ying and Simmons 2020). Adipokine and cytokine synthesis are dysregulated, and lipid storage is hampered by adipose tissue malfunction (Ou et al. 2022). As people age, they are more likely to develop metabolic disorders like diabetes and metabolic syndrome. This is partly due to the fact that when humans and other animals age, the cells in adipose and other tissues lose their sensitivity to insulin (Tchkonia, Thomou, et al. 2013). Additionally, as people age, preadipocytes that produce new, insulin‐sensitive adipocytes malfunction. This has to do with the buildup of senescent cells, which secrete SASP that are harmful to both surrounding and distant cells in contrast to typical preadipocytes (Tchkonia, Zhu, et al. 2013).

In this study, we showed that MEFs and adipose tissue from MKP‐2‐deficient mice are resistant to premature senescence. Targeted mass spectrometry‐based lipidomic assays revealed increased levels of hexosylceramides (HexCER) and phosphatidylglycerols (PG) and reduced levels of diacylglycerols (DGs) in MKP‐2‐deficient mouse adipose tissues. We identify adipose lipid remodeling as a potential component of the mechanism by which MKP‐2 deficiency promotes metabolic health and longevity. These findings suggest that MKP‐2 regulates lipid metabolism in adipose tissue, with potential implications for adipose tissue development and biological aging.

## 2 Materials and Methods

### 2.1 Reagents and Antibodies

All reagents were purchased from standard chemical vendors. The following antibodies were used: phospho‐p38 MAPK (#4511S), phospho‐JNK1/2 (#4668S), p38 MAPK (#9228 s), JNK (#3708 s), phospho‐Akt (Ser473) (#9217), phospho‐Akt (Thr308) (#9275), α‐tubulin (#2125), β‐actin (#8457), lamin B1 (#13435), HSP90 (#4874), phospho‐histone H2A.X (Ser139) (#9718), p21 (#2947), p16 (#80772), PAI‐1 (11907), and pp53 (#9284). All antibodies were obtained from Cell Signaling Technology. Adipose tissues from MKP‐2 wild‐type (WT) and MKP‐2 KO mice were isolated, processed, and immunoblotted as described (Fernando et al. 2022).

### 2.2 Animal Studies

The University of Alabama in Huntsville Institutional Animal Care and Use Committee approved all animal studies (Fernando et al. 2022). The MKP‐2 global MKP‐2 KO (MKP‐2 KO) mice were kindly provided by Dr. Robin Plevin, University of Strathclyde, United Kingdom. MKP‐2 KO mice have already been described genetically and metabolically (Al‐Mutairi et al. 2010; Fernando et al. 2022). Survival analysis: Throughout the study, mouse survival was tracked, and the Kaplan–Meier method was used to create survival curves (Mantel 1966). The log‐rank (Mantel–Cox) test was used to evaluate differences between experimental groups (Mantel 1966). Statistical significance was defined as a *p* < 0.05.

### 2.3 Culture of Mouse Embryonic Fibroblasts and Transient Transfections

The isolation and culture of MEFs have been described previously (Lawan et al. 2011). MKP‐2 KO and WT mice were sacrificed when embryos were between 12 and 14 days old. The cells were cultured in Dulbecco's modified Eagle's medium (DMEM) supplemented with 10% FBS, penicillin, and streptomycin, at 37°C and 5% CO_2_. The mammalian expression vectors pSRa‐Flag‐MKP‐2 expressing wild type MKP‐2 have been previously described (Crowell et al. 2014). MEFs were transiently transfected with 5 ug of Flag‐MKP‐2 for 48 h using lipofectamin 3000 according to manufacturer's instruction (Fisher Scientific; Cat #L3000015).

### 2.4 Senescence‐Associated β‐Galactosidase Staining and Imaging of MEFs and Adipose Tissue

Cellular senescence in MEFs was assessed using a senescence‐associated β‐galactosidase (SA β‐gal) staining kit (Cell Signaling Technology) according to the manufacturer's instructions. After fixing the cells, SA β‐gal activity was found after treating cells with X‐gal staining solution for 8–12 h at 37°C at pH 6.0 in a dry incubator without CO_2_. Images of the cells were taken at 10× resolution using the Seba Digital Imaging System microscope (LAXCO Inc., Bothell, WA) to count the total cells, the cells that were stained, and cells that had an altered morphology, with the stain becoming blue in the presence of SA β‐gal activity (Hamsanathan and Gurkar 2022). Data were processed by collecting the number of total cells, the number of cells showing the blue stain, and the number of cells showing morphological changes associated with senescence. Altered morphology included flattening of the cell and the presence of multiple nuclei (He and Sharpless 2017). In adipose tissues, SA‐β‐Gal was measured as described (21). 19‐month‐old WT and MKP‐2 KO male mice were fed a custom high‐fat (60% kcal) purified rodent diet DN112252 (Dyets Inc., Bethlehem, PA, USA) for ~4–6 months. Adipose tissues were collected and fixed for five minutes in PBS containing 0.25% glutaraldehyde (Sigma‐Aldrich) and 2% (vol/vol) formaldehyde (Sigma‐Aldrich) after being rinsed three times with PBS. After fixation, tissues were rinsed three times with PBS and then incubated for 16–18 h at 37°C in SA‐βGal activity solution (pH 6.0). Tissues were washed three to five times with ice‐cold PBS to halt the enzymatic activity. Imaging was done using the Laxco Seba2 Digital Microscope (Laxco Inc.). In each sample, eight to ten images1 were obtained from arbitrary fields.

### 2.5 Telomere Activity Quantification With qPCR Analysis

Lysates from MEFs derived from MKP‐2 WT and MKP‐2 MKP‐2 KO mice were used to measure telomerase activity using the Sciencell Telomerase Activity Quantification qPCR Assay kit (ScienCell Research Laboratories, CA) according to the manufacturer's instructions. Real‐time quantitative PCR was used to measure telomere length as described (Fernando et al. 2022), with the Applied Biosystems 7500 Fast RT‐PCR system.

### 2.6 RNA Extraction and Real‐Time PCR Analysis

Cells and tissues derived from MKP‐2 WT and MKP‐2 MKP‐2 KO mice were used to isolate total RNA using E.Z.N.A HP kit (Omega Bio‐Tek Inc. GA) according to the manufacturer's instructions and as described (Fernando et al. 2022). Real‐time quantitative PCR was performed as described (Fernando et al. 2022), with the Applied Biosystems 7500 Fast RT‐PCR system and primers for SYBR Green Gene Expression Master Mix listed in Table 1. All relative gene expression levels were analyzed using the ΔCt method and normalized to 18S.

**TABLE 1:** List of primer sequences used for qPCR analysis.

Name	Forward	Reverse
p21	CCTGGTGATGTCCGACCTG	CCATGAGCGCATCGCAATC
p16	CGCAGGTTCTTGGTCACTGT	TGTTCACGAAAGCCAGAGCG
Cers6	GGAATGGCCTTGTTCGGCT	CTGAGTCGTGAAGACAGAGG
IL‐6	TAGTCCTTCCTACCCCAATTTCC	TTGGTCCTTAGCCACTCCTTC
p53	TCACAGCGTCTGTTGACATTT	ACCAAGCTCATTACCCTGACA
18S	ACCGCAGCTAGGAATAATGGA	GCCTCAGTTCCGAAAACCA
DGAT1	GACGGCTACTGGGATCTGA	TCACCACACACCAATTCAGG
PGS1	GTTCCTCAGGTCACCTCCC	CCATCACAACTCGCCTCTTGG
PGPP	ACTATGAACGAGGAGTACGAGAC	GTTGGGACCCCAGTCATGTC
TNFα	CCCTCACACTCAGATCATCTTCT	GCTACGACGTGGGCTACAG
CCL2	TTAAAAACCTGGATCGGAACCAA	GCATTAGCTTCAGATTTACGGGT

### 2.7 Immunoblotting

Cells and adipose tissue were homogenized in RIPA buffer (25 mM Tris. HCl pH 7.4, 150 mM NaCl, 5 mM EDTA, 1% NP‐40, 0.1% SDS, and 1.0% sodium deoxycholic acid), supplemented with protease and phosphatase inhibitors (5 μg/mL leupeptin, 5 μg/mL aprotinin, 1 μg/mL pepstatin A, 1 mM PMSF, 1 mM benzamidine, 1 mM Na_3_VO_3_, and 10 mM NaF). Homogenates were lysed for 30 min on a shaker and then clarified at 20,800 *g* for 30 min at 4°C. The Pierce BCA Protein Assay Kit (Pierce, Rockford, IL) was used to measure the protein concentrations. Lysates were separated using SDS‐PAGE, then transferred onto nitrocellulose membranes, and treated with phospho‐specific antibodies which were detected by enhanced chemiluminescence.

### 2.8 Quantitative Lipidomics Sample Preparation

Lipids were extracted from previously weighted (range of 13.3–201.9 mg) frozen adipose tissue samples in two batches of 33 samples at a time. Samples were thawed at 4°C for 30 min, 100 μL of deionized H_2_O was added to each sample, and samples were homogenized in a bullet blender for 10 min. 100 μL of each sample was transferred to 2 mL Eppendorf PCR‐clean vials. 25 μL spiking solution containing 69 isotope‐labeled lipid internal standards, 75 μL H_2_O, 575 μL methyl tertiary butyl ether (MTBE), and 160 μL methanol were added, and the solution was vortexed for 10 s. Samples were then shaken at 21°C and 500 rpm for 30 min, 200 μL H_2_O was added, and samples were centrifuged at 14,000 rpm for 3 min at 21°C. The upper layer was collected into a new disposable 14 × 100 mm glass culture tube. A second extraction was performed on the residual sample that remained in the 2 mL Eppendorf vials by adding 300 μL MTBE, 100 μL methanol, and 100 μL H_2_O, shaking for 5 min at 21°C and 500 rpm, centrifuging at 14,000 rpm for 3 min at 21°C, and transferring the new upper layer to merge it with the previously collected upper layer in the glass tubes. Finally, the samples were dried at 30°C for 30 min in a Biotage N_2_ drier, reconstituted in 200 μL of 10 mM ammonium acetate in 50/50 methanol/methylene chloride, and transferred to auto‐sampler glass vials for lipidomics analysis.

### 2.9 Quantitative Lipidomics Mass Spectrometry Data Acquisition

Lipid species were measured using a Sciex Lipidyzer system (Sciex, Framingham, MA) as described elsewhere (Su et al. 2021). The mass spectrometry (MS) platform consisted of Shimadzu Nexera X2 LC‐30 AD pumps, a Shimadzu Nexera X2 SIL‐30 AC autosampler kept at 10°C, and a Sciex QTRAP 5500 mass spectrometer equipped with an electrospray ionization (ESI) source coupled with SelexION for differential mobility spectrometry (DMS). 1‐propanol was used as the chemical modifier for the DMS. Samples were introduced to the MS by flow injection at 8 μL min^−1^. Each sample was injected twice: first with the DMS ON (for lipid classes: PC, PE, LPC, LPE, PG, PI, PS, and SM, Table 2), and then with the DMS OFF (for classes: CE, CER d18:0, CER d18:1, DAG, FFA, HexCER, LacCER, LPG, LPI, LPS, PA, and TAG, see Table 2).

**TABLE 2:** Abbreviation of lipid classes.

Abbreviation	Lipid class
CE	Cholesterol Ester
Cer d18:0	Ceramides
Cer d18:1	Ceramides
DG	Diacylglycerol
FFA	Free fatty acids
HexCER	Hexosylceramides
LPC	Lysophosphatidylcholine
LPE	Lysophosphatidylethanolamine
LPG	Lyso‐phosphatidylglycerol
LPI	Lysophosphatidylinositol
LPS	Lipopolysaccharides
LacCER	Lactosylceramide
PA	Phosphatidic acid
PC	Phosphatidylcholine
PE	Phosphatidylethanolamine
PG	Phosphatidylglycerol
PI	Phosphoinositides
PS	Phosphoserine
SM	Sphingomyelin
TG	Triacylglycerol

Across 20 lipid classes/sub‐classes, 1561 species (plus 69 spiked isotope‐labeled lipid standards) were targeted using multiple reaction monitoring and positive/negative polarity switching ESI. The species in classes SM, DAG, CE, CER d18:0, CER d18:1, HexCER, LacCER, LPG, LPI, LPS, PA, LPE, LPC, and TAG were ionized in positive and in classes PE, PG, PI, PS, PC, and FFA in negative ionization mode, respectively. Data were acquired using Sciex Analyst 1.6.3 and processed with Shotgun Lipidomics Assistant v4.1.2 Python software (Su et al. 2021). For quality control, an in‐house pooled human serum sample was run at the beginning and at the end of each of two 33‐sample batches.

### 2.10 Quantitative Lipidomics: Data Analysis

Statistical analysis was conducted in R (R Core Team 2022). Where applicable, we used false discovery rate (FDR) correction for multiple testing (Benjamini and Hochberg 1995). Among 1561 species from 20 classes, 324 were detected in at least 90% of samples and the remaining species were removed. The data were log_e_‐normalized, and the effects of batch were removed by taking residuals of least squares regression of each species on batch. To adjust for sample‐to‐sample variation, the data were then mean‐centered by sample. Of the 324 normalized lipids, there were 224 with up to 10% missing values, and these missing values were imputed by 10‐nearest neighbor mean imputation. We used principal components analysis (PCA) on scaled species data to identify three outlier samples (Mahalanobis distance [across PC_1‐2_], of > 2 SD). These samples were three of the six taken from brown adipose tissue of 6 month old WT mice and were removed, leaving normalized data for 324 species in 63 samples.

Principal components analysis was applied to the normalized species data to depict sample clustering by tissue, genotype, and age. To estimate the main and interaction effects of genotype (*G*, WT or MKP‐2 MKP‐2 KO), by age (as a factor: 6, 12 and 14–16 months, A), and by tissue (*T*, BAT or EWAT) on each lipid species (Y), we fit a linear model that also included sample weight (wt) as a covariate (Equation 1):

(1) $$Y=x+\beta_{G}+\beta_{A}+\beta_{T}+\beta_{\mathrm{GxA}}+\beta_{\mathrm{AxT}}+\beta_{\mathrm{GxT}}+\beta_{\text{GxAxT}}+\beta_{\mathrm{wt}}+\varepsilon$$

The significance of each term was tested for by ANOVA and adjusted for multiple testing by FDR (Korthauer et al. 2019). For lipid class‐level analysis, we asked if the regression coefficients for the effects of genotype ($\beta_{G}$) among all species in a class (*n* = 3–97 species per class, C), were different from zero using a one sample *t*‐test.

(2) $$\beta_{G}=0+\beta_{C}+\varepsilon$$

## 3 Results

### 3.1 MKP‐2 Deletion Reduced Cellular Senescence in MEFs

MKP‐2 KO mice have already been described genetically and metabolically (Al‐Mutairi et al. 2010; Fernando et al. 2022). One crucial strategy for preserving tissue homeostasis is the control of cell division and senescence. We have previously demonstrated that MKP‐2 is crucial for cell survival and proliferation (Lawan et al. 2011). To investigate the function of MKP‐2 in cellular senescence in primary MEFs, SA β‐gal activity was assessed in MEFs derived from WT and MKP‐2 KO mice at the same passage. When compared to MKP‐2 KO MEFs, MKP‐2 WT MEFs showed a flattened and expanded senescence‐like shape with considerably higher β‐gal activity (arrows in Figure 1A–C). In contrast, MKP‐2 KO MEFs exhibited reduced β‐gal activity compared with WT controls (Figure 1A–C). These results suggest that MKP‐2 is important in the development of premature senescence.

**FIGURE 1:**
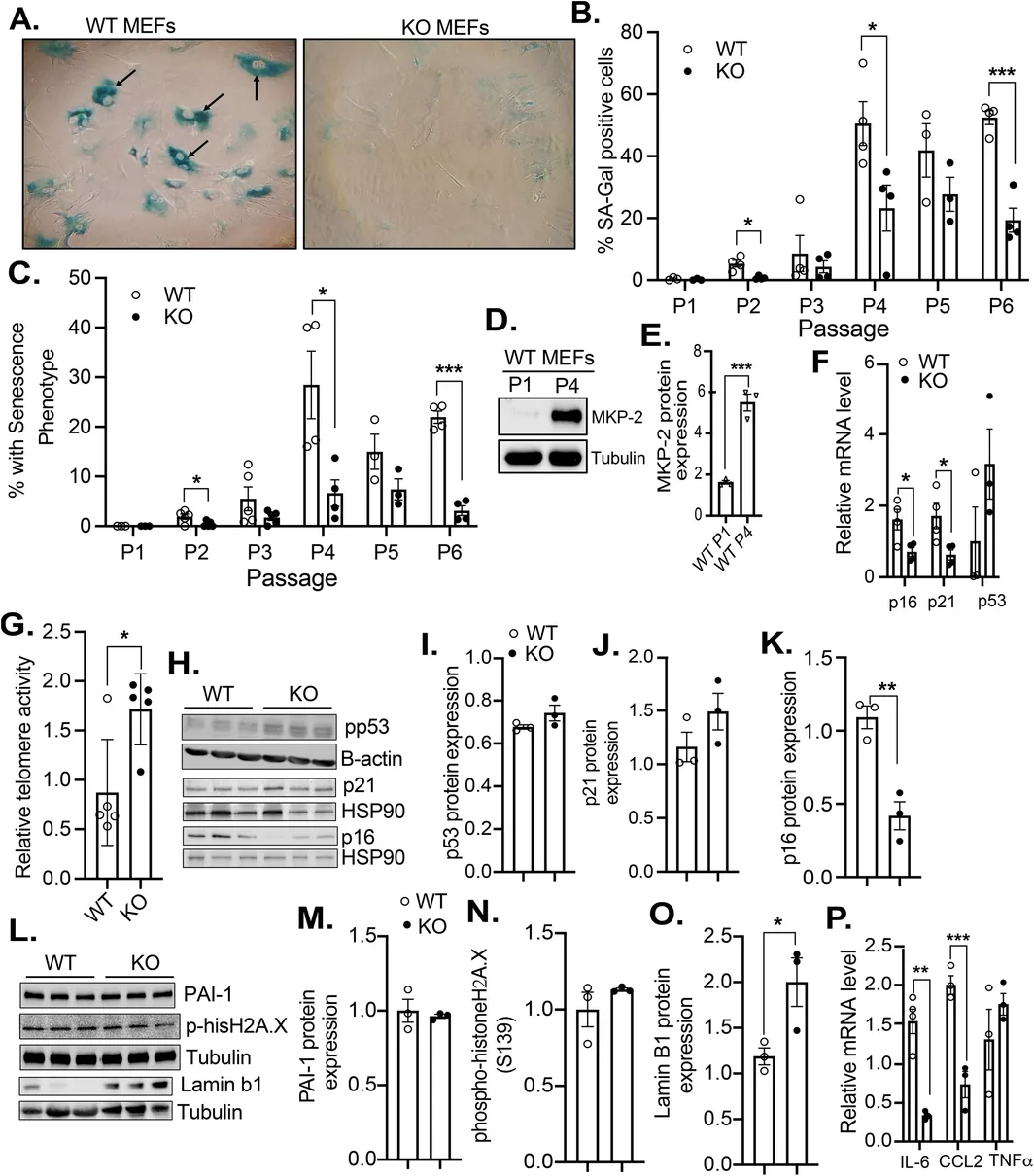
MKP‐2 Deletion Reduced Cellular Senescence in MEFs. (A) Representative images of senescence‐associated β‐galactosidase staining. (B, C) Percentage of β‐gal positive cells and associated phenotype (*n* = 6). (D, E) Wild type MEFs (passage 1 and 4) were used to assess MKP‐2 protein expression by immunoblotting using specific antibodies. Immunoblots were quantitated by densitometry for the levels of MKP‐2 and normalized to Tubulin, mRNAs for (F) p21, p16, and p53 (G) Relative telomere length was measured in P4 from WT and KO MEFs (*n* = 4). (H–K) Immunoblots for phospho‐p53, p21, and p16 from MEFs lysates were quantitated by densitometry and normalized to β‐actin and HSP90. (L–O) Immunoblots for PAI‐1, p‐hisH2A.X, and lamin B1 from MEFs lysates were quantitated by densitometry and normalized to α‐tubulin (*n* = 3–4). (P) mRNAs for IL‐6, CCL2, and TNFα were assessed by QPCR MEFs (*n* = 3–4). Mean ± SEM. *p < 0.05; **p < 0.01; ***p < 0.0001; as determined by *t*‐tests.

To investigate whether MKP‐2 protein level changes in senescent WT MEFs, we assessed MKP‐2 protein expression in early (P1) and senescent MEFs (P4). We found that MKP‐2 protein levels significantly increased in senescent WT MEFs compared with the non‐senescent cells (Figure 1D,E). These results show that MKP‐2 is more abundant in later passages in premature senescent cells.

### 3.2 Senescence‐Associated Increased Lamin B1 Expression in MKP‐2 KO MEFs

To further investigate the molecular mechanism of reduced senescence in MKP‐2 KO MEFs, quantitative PCR analysis of mRNA expression of p21 and p16 was performed in MEFs derived from MKP‐2 WT and MKP‐2 KO mice. Research has demonstrated that MAPKs regulate p21 and p16, which are crucial for controlling cellular senescence (Moosavi and Yazdanparast 2008; Mirzayans et al. 2012). Consistent with protection from senescence in MKP‐2 KO MEFs, the expression of mRNAs for p21 and p16 was significantly reduced in MKP‐2 KO MEFs but not p53 expression (Figure 1F). Studies have shown that telomere shortening can be linked to earlier senescence (Chen, Tang, et al. 2019). To determine if telomere length may contribute to the MKP‐2 KO MEFs' resistance to senescence, a relative telomere length assay was performed on genomic DNA from MEFs derived from the MKP‐2 KO and WT controls. We found that the relative telomere length of the MKP‐2 KO MEFs was significantly longer compared with WT MEFs (Figure 1G). The p53 pathway is important to cellular senescence (Regulski 2017). When comparing MKP‐2 KO MEFs to WT MEFs, there was no discernible difference in p53 phosphorylation (Figure 1H,I). There was no change in protein expression as a result of the decreased p21 mRNA (Figure 1F,H–J). However, we found decreased p16 expression in MKP‐2 KO MEFs compared with WT MEFs (Figure 1H,K). Next, we examined the expression of plasminogen activator inhibitor‐1 (PAI‐1), a useful senescence marker. When comparing MKP‐2 KO MEFs to WT MEFs, there was no difference in PAI‐1 expression (Figure 1L,M). There was no difference in gamma‐H2A.X (γH2A.X) phosphorylation at Ser139 between WT and MKP‐2 KO MEFs (Figure 1L,N). Loss of lamin B1 is associated with the senescence phenotype. Consistent with senescence protection in MKP‐2 KO MEFs, we found increased lamin B1 expression in MKP‐2 KO MEFs compared with WT cells (Figure 1L,O). Senescent cells influence their surroundings via senescence‐associated secretory phenotype (SASP) (Tchkonia, Zhu, et al. 2013). We found reduced expression of IL‐6 and CCL‐2 in MKP‐2 KO MEFs, but not TNFα expression compared with WT cells (Figure 1P). These data demonstrate that MKP‐2 deletion suppresses premature senescence and increases lamin B1 expression without substantially altering γH2A.X response.

### 3.3 MKP‐2 Is Required for Premature Senescence in MEFs

Having established that MKP‐2 deficiency protects against premature senescence, we conducted gain‐of‐function studies. Overexpression of flag‐tagged WT MKP‐2 in MKP‐2 KO MEFs effectively restored the senescence phenotype similar to those in WT MEFs (Figure 2A–D). These results indicate that MKP‐2 deficiency protects against accumulation of senescent cells, and MKP‐2 has a direct role in the development of premature senescence.

**FIGURE 2:**
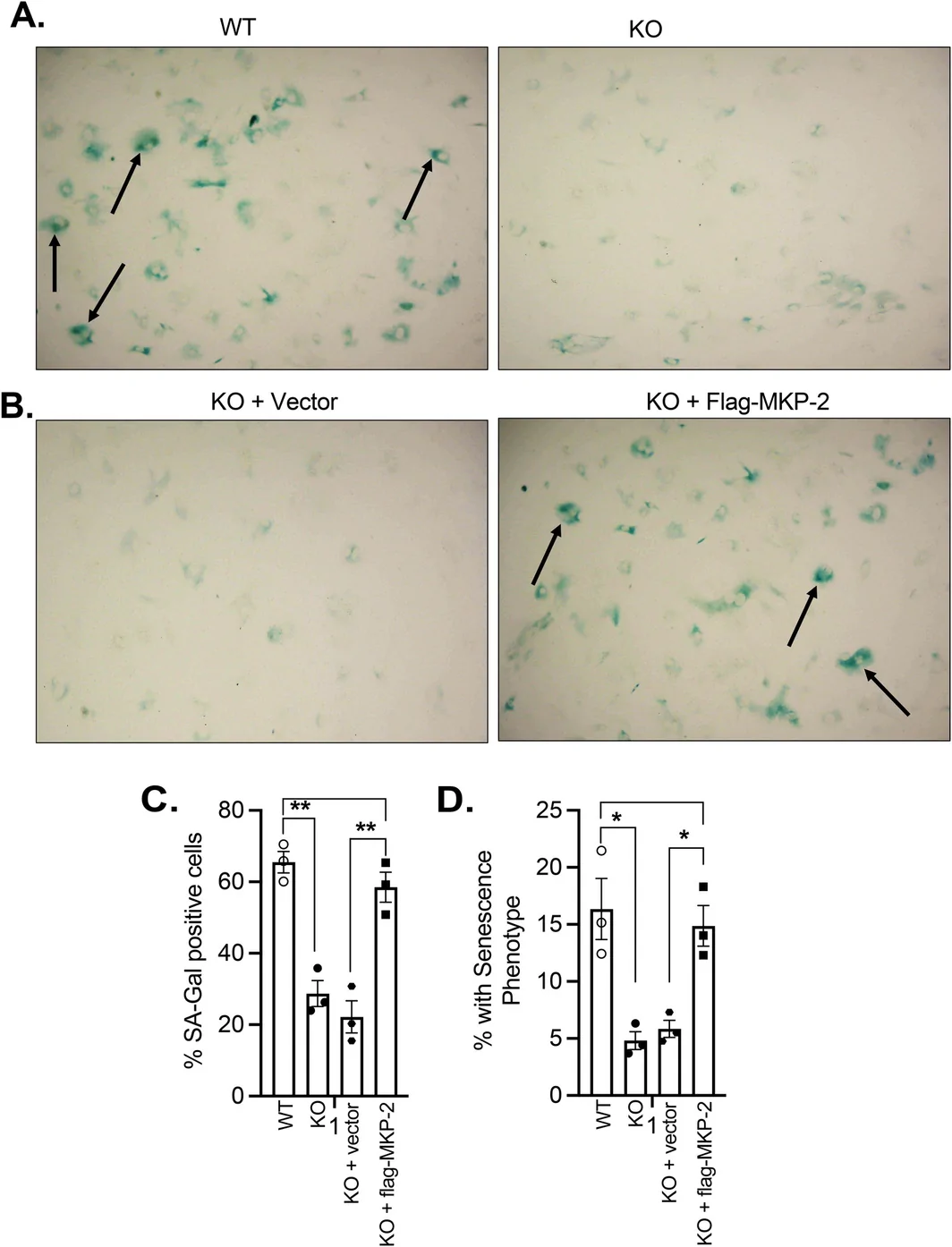
MKP‐2 is required for MEF senescence. (A, B) Representative images of senescence‐associated β‐gal staining. (C, D) Percentage of β‐gal positive cells and associated phenotype (*n* = 3). WT and KO MEFs were transfected with vector or flag‐MKP‐2. Mean ± SEM. *p < 0.05; **p < 0.01; as determined by *t*‐tests.

### 3.4 MKP‐2 Deficiency Reduced Senescence in Adipose Tissue

Expanding on the knowledge of MKP‐2's function in MEF senescence, we hypothesize that MKP‐2 deficiency protects against senescence in metabolic organs. White adipose tissue (WAT) experiences substantial remodeling with aging, characterized by changes in fat distribution, an increase in visceral fat, decreased thermogenesis, and persistent low‐grade inflammation that dysregulates immunological and metabolic homeostasis (Czech 2020). New findings from single‐cell and spatial transcriptomics emphasize the crucial roles that adipocyte senescence plays in causing systemic dysfunction that leads to age‐related diseases (Hamsanathan and Gurkar 2022). Additionally, WAT is characterized by higher inflammation and senescence compared to other fat depots (Czech 2020). To determine the senescence status in vivo, we assessed senescence in epididymal white adipose tissue (EWAT) derived from high‐fat diet (HFD)‐fed male MKP‐2 KO and WT mice. Assessment of SA‐β‐gal activity in chow‐fed mice is a challenge. We found that EWAT from 19‐month‐old HFD‐fed MKP‐2 KO mice exhibited a reduced amount of SA‐β‐gal activity compared with WT mice (Figure 3A,B). These results indicate that MKP‐2 deficiency protects against accumulation of senescent cells in adipose tissue. To explain these differences in senescence, we examined several targets linked to senescence. We measured the phosphorylation status of p53 in EWAT of 19‐month‐old HFD‐fed MKP‐2 KO mice. MKP‐2 KO mice displayed decreased levels of EWAT p53 phosphorylation (Ser15) as compared with WT controls (Figure 3C,D). Similarly, MKP‐2 KO mice exhibited reduced levels of EWAT p16, but not p21, expression compared with WT mice (Figure 3C–E). Next, we examined PAI‐1 expression in EWAT. MKP‐2 KO mice showed reduced PAI‐1 expression in comparison to WT mice (Figure 3G,H). However, histone H2A.X EWAT phosphorylation (Ser139) did not differ between the MKP‐2 KO and WT mice (Figure 3G,I). Remarkably, we discovered that MKP‐2 KO EWAT expressed more lamin B1 than WT EWAT (Figure 3G,J). These findings show that MKP‐2 deletion increases lamin B1 expression, decreases p53 phosphorylation, and reduces stress‐induced premature senescence in EWAT, without significantly changing the H2A.X response. Senescent cells influence their surrounding cells via SASP (He and Sharpless 2017). We evaluated SASPs in EWAT. In comparison to controls, the EWAT of HFD MKP‐2 KO mice showed significantly lower levels of mRNA expression for p53, IL‐6, p16, and p21 compared with WT mice (Figure 3K–N). Additionally, we used a mouse cytokine antibody array to measure the amounts of mouse proteins in the blood serum of MKP‐2 KO and WT mice that were fed a HFD in order to assess the potential that MKP‐2 controls the expression and secretion of SASP components. Changes in the protein composition of MKP‐2 mice were shown by densitometric analysis. We discovered an overrepresentation of a certain set of SASP molecules. IL‐6 and IL‐15 were among those that demonstrated statistically lower levels in MKP‐2 KO animals in comparison to control mice (Figure 3O,P). To determine how MKP‐2 regulates MAPKs, we measured the phosphorylation status of JNK and ERK in the EWAT of MKP‐2 KO mice, which exhibited increased phosphorylation levels of both JNK and ERK compared with WT mice (Figure 3Q–S). These findings support the notion that MKP‐2 deficiency prevents stress‐induced premature senescence, pointing to a possible involvement of MKP‐2 in the onset of EWAT senescence. It also demonstrates that MKP‐2 plays a vital role in negatively regulating JNK and ERK.

**FIGURE 3:**
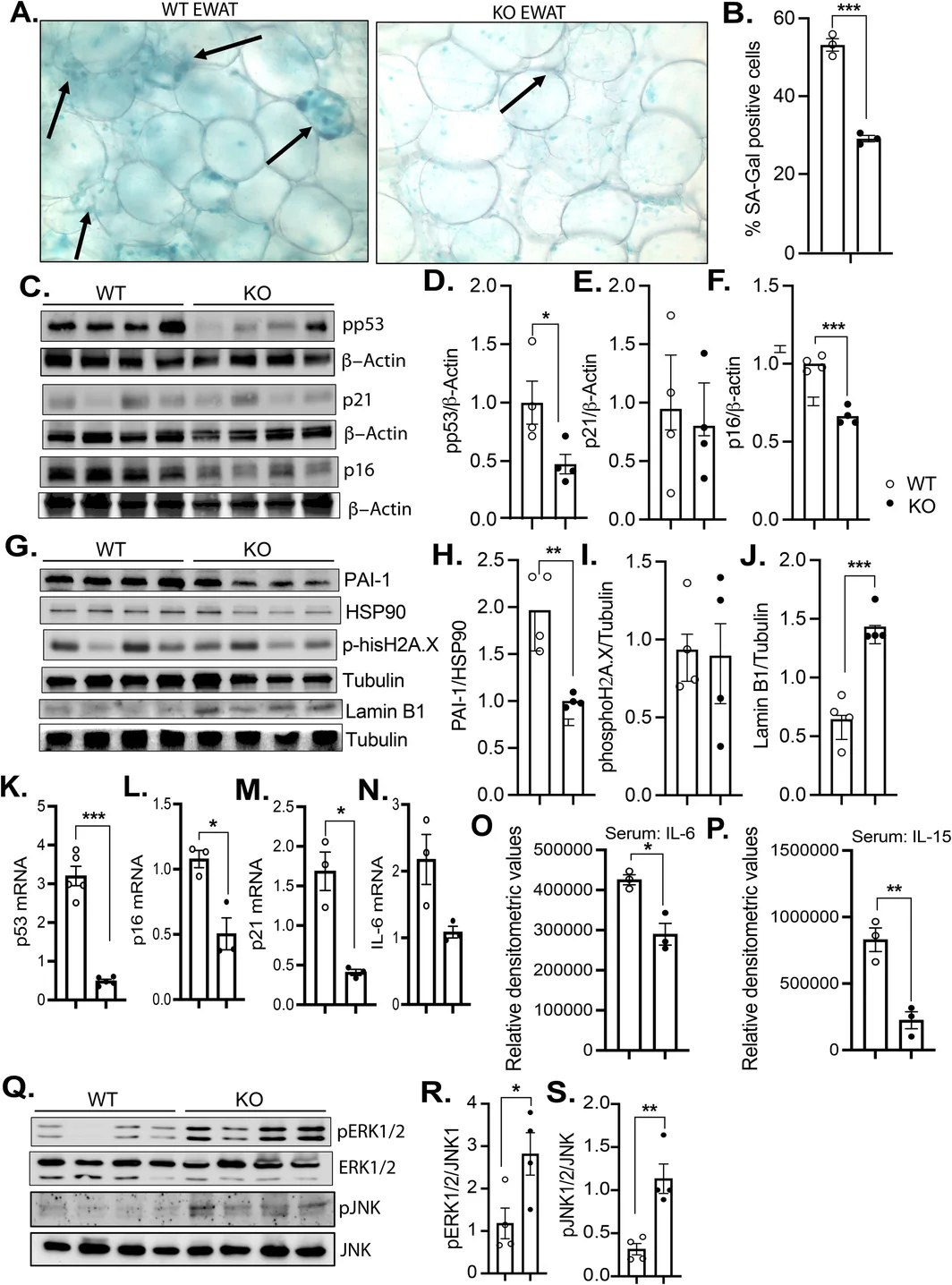
Reduced premature senescence in white adipose tissue of HFD‐fed MKP‐2 KO. (A, B) Representative images of senescence associated β‐gal staining and percentage of β‐gal positive cells in EWAT from 19‐month old male KO mice fed a HFD for 4–6 months (*n* = 3). (C–F) Immunoblots for phospho‐p53, p21, and p16 from EWAT lysates were quantitated by densitometry and normalized to β‐actin. (G–J) Immunoblots for PAI‐1, p‐hisH2A.X, and lamin B1 from EWAT lysates were quantitated by densitometry and normalized to HSP90 and α‐tubulin (*n* = 3–4). mRNAs for (K) p53 (L) p16 (M) p21 (N) IL‐6 were assessed by QPCR from EWAT (*n* = 3–5), and (O, P) serum protein levels of secreted cytokines (*n* = 3) of HFD‐fed male WT and KO mice for 24‐weeks. White adipose tissue lysates from HFD‐fed mice were analyzed by immunoblotting. Immunoblots were quantitated by densitometry for the levels of phospho‐JNK1/2 and ERK1/2 (Q–S) normalized to corresponding total MAPKs (*n* = 4–6). Mean ± SEM. *p < 0.05; **p < 0.01; ***p < 0.0001; as determined by *t*‐tests.

### 3.5 MKP‐2 KO Affects the Abundance of Hexosylceramide, Phosphatidylglycerol, and Diacylglycerol in Adipose Tissue

The observation that EWAT from MKP‐2 KO mice is protected against senescence prompted us to examine the mechanisms that link MKP‐2 signaling and adipose tissue senescence. Furthermore, we have previously demonstrated that MKP‐2 KO mice eat less food and have a lower respiratory exchange ratio compared with WT controls, which suggests that they are burning more lipids from their fat stores (Fernando et al. 2022). Also, HFD‐fed MKP‐2 KO mice exhibit reduced fat mass and smaller adipocytes compared with WT mice (Fernando et al. 2022). And so, we asked how lipid composition may be altered in MKP‐2 KO mice. We conducted targeted mass spectrometry‐based lipidomic assays (over 1500 lipids across 20 lipid classes/subclasses) in mouse adipose tissue of 6‐, 12, and 14–16‐month‐old male MKP‐2 KO and WT mice in order to identify which lipid species alterations are caused by MKP‐2 deletion and linked to changes in lipid metabolism. We detected 324 lipid species among EWAT and BAT of MKP‐2 KO and WT mice.

We then tested for effects of genotype, age, tissue, and their interaction on each lipid species, and we identified 12 species with effects of genotype (ANOVA, FDR ≤ 0.05, Figure 4A) and 28 species with genotype by tissue interaction effects (FDR ≤ 0.05, Figure 4B). The species with either effects of genotype included lipids from four classes, and those of genotype by tissue interactions included lipids from four and seven lipid classes, respectively, as well as four of the free fatty acids (Figure 4B).

**FIGURE 4:**
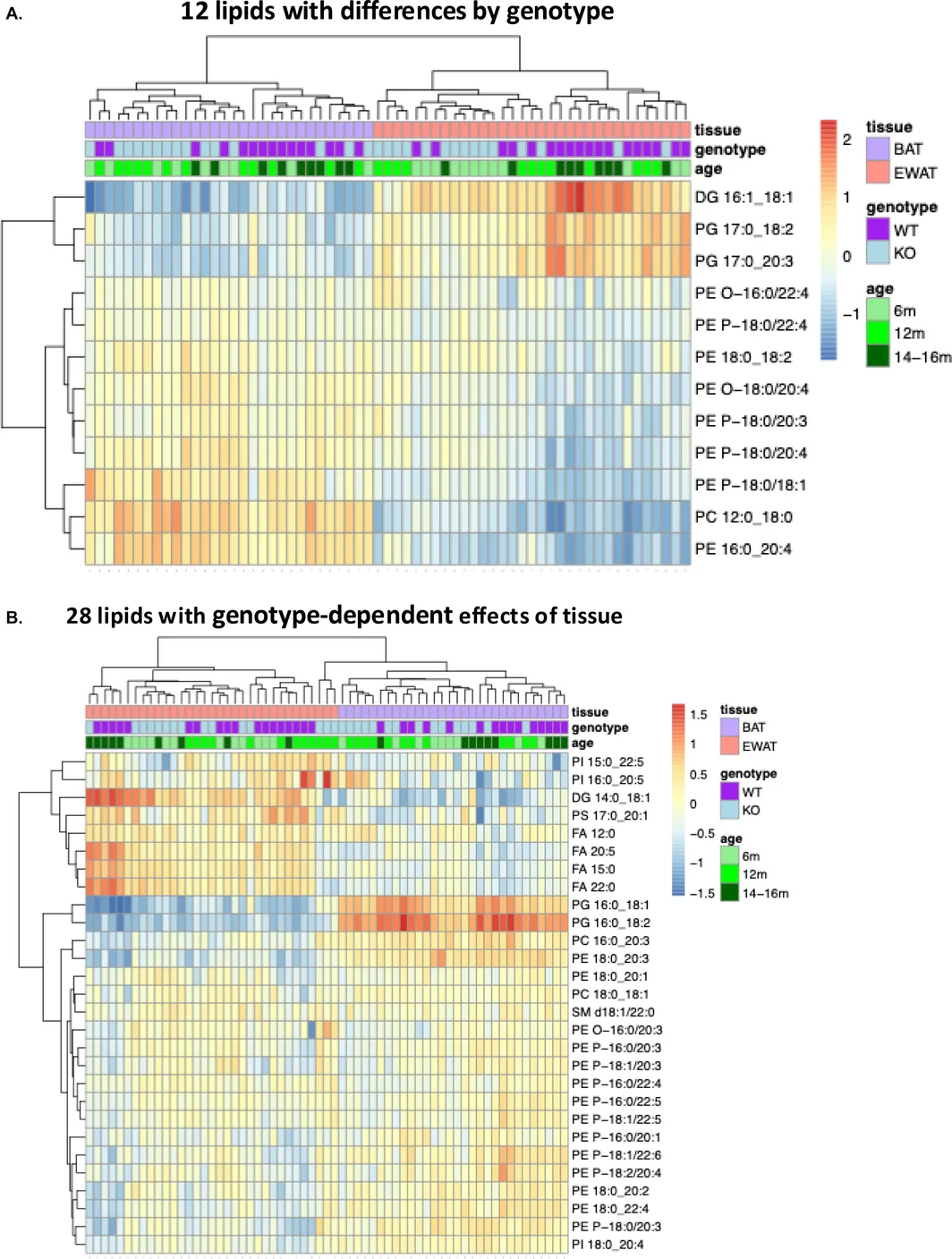
Heat map of lipids with effect of genotype and lipids with genotype‐dependent effects of BAT and EWAT in targeted lipidomic analysis of male MKP‐2 KO mice. (A) Heat map of the 12 different lipid species from BAT or EWAT with effect of genotype (FDR ≤ 0.05) in 6‐, 12‐, and 14–16‐month‐old MKP‐2 KO compared with WT (*n* = 6 mice/genotype/age group). (B) Heat map of the 28 different lipid species with genotype‐dependent effects of BAT and EWAT (FDR ≤ 0.05) in 6‐, 12‐, and 14–16‐month‐old MKP‐2 KO compared with WT mice. Color scale in A and B representing *Z*‐scores.

To assess whether MKP‐2 KO affects the abundance of any lipid classes, we looked for over‐represented among the species with genotype effects and then asked if the abundance of any class was different by genotype. We did not find any lipid classes enriched by over‐representation among the individual species that associated with genotype (Fisher's test, *p* > 0.05). However, when the regression coefficients of each species on genotype ($\beta_{G}$, Methods) were aggregated by class, those among species of hexosylceramides (HexCER) and phosphatidylglycerols (PG) were significantly greater than zero, indicating that lipids in these classes are more abundant in the adipose tissue of MKP‐2 KO mice (Figure 5A). Conversely, coefficients for DGs were less than zero indicating that DGs are less abundant in the adipose tissue of MKP‐2 KO mice (Figure 5A). This analysis evaluated effects of genotype (*β*_*G*_) while controlling for age and tissue (Methods), and does not also resolve interaction effects of genotype, such as by age or by tissue. HexCERs, a type of glycosphingolipid, are made up of a hydrophilic hexose (glucose or galactose) connected to a hydrophobic ceramide (Cer) backbone (Hannun and Obeid 2018). Among the HexCER were HexCER d18:1/22:0, HexCER d18:1/22:1, and HexCER d18:1/24:0 (Table S1). Among the PGs were PG 16.0_18.1 and 16.0_18.2 (Table S1). Diacylglycerol is a lipid made up of two fatty acids attached by ester linkages to positions 1 and 2 of glycerol (Carrasco and Mérida 2007). Among the DGs were DG 16:0_18:3, DG 18:1_18:2, DG 18:2_18:3, DG 14:0_18:1, and DG 14:0_18:2 (Table S1). Triacylglycerol (TG) which is stored in lipid droplets, can be produced using DG (Topham and Epand 2009). Consistent with reduced DGs, we previously showed that at 6 months of age, MKP‐2‐deficient mice had lower hepatic TGs (Fernando et al. 2022). These lipidome data suggest that the quantities of HexCER, PGs, and DGs in adipose tissue are impacted by MKP‐2 deficiency, suggesting that MKP‐2 might be involved in the regulation of these lipid classes.

**FIGURE 5:**
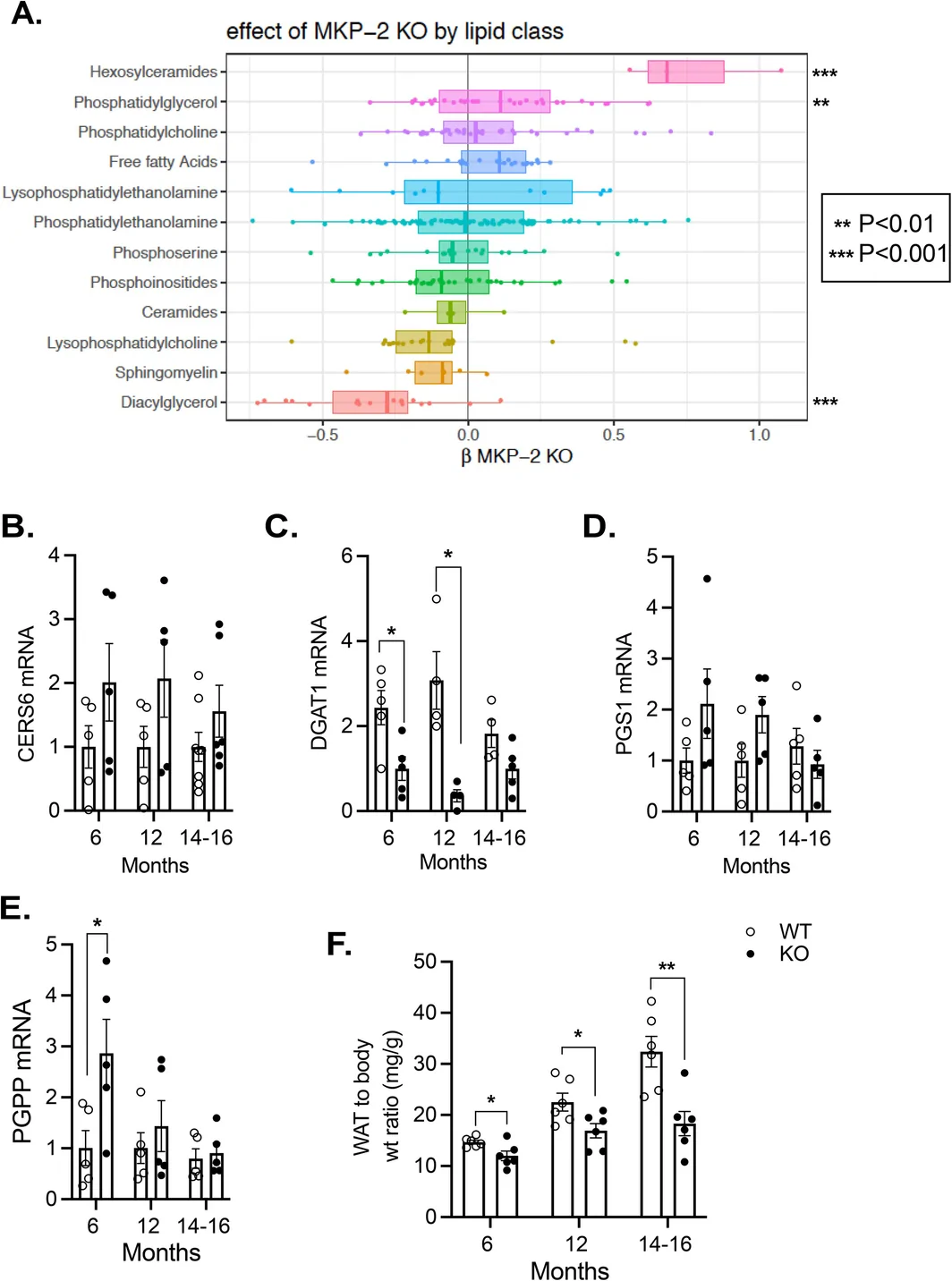
Enhanced hexosylceramides and phosphatidylglycerols and reduced diacylglycerols in targeted lipidomic analysis of BAT and EWAT of aged male MKP‐2 KO mice. (A) The regression coefficients for genotype on each lipid species. (β MKP‐2 KO) are plotted by lipid class (*n* = 3–97 species per class). Positive values of β MKP‐2 KO are lipids that are higher in the mutant and negative, lower. The β MKP‐2 KO by lipid class were tested for differences from zero, and two classes hexosylceramides and phosphatidylglycerols were greater than zero, and diacylglycerols were less than zero (*p* < 0.05). mRNAs for (B) Cers6, (C) DGAT1, (D) PGS1, (E) PGPP from EWAT, and (F) EWAT mass to body mass ratio in 6‐, 12‐, and 14–16‐month‐old MKP‐2 KO and WT mice (*n* = 5–6 mice/genotype/age group). Bars in B through F show the mean ± SEM. *p < 0.05; **p < 0.01; ***p < 0.0001; as determined by *t*‐tests.

To establish if these changes in adipose tissue are due to transcriptional changes, we measured expression of ceramide synthase 6 (Cers6) by RT‐qPCR in the adipose tissue of a separate cohort of 6‐, 12‐ and 14–16‐month‐old MKP‐2 KO and WT mice. Even though the lipidomic study was done in both EWAT and BAT, we concentrate all of our analysis on EWAT since it is the depot most vulnerable to senescence. Expression of Cers6 tended to be higher in all of the age groups in MKP‐2 KO mice compared with WT controls (Figure 5B), however, this was not statistically significant. When compared to WT controls, we observed significantly lower triglyceride synthase enzyme (DGAT1) expression in 6‐ and 12‐month‐old MKP‐2 KO, but not 14–16‐month‐old MKP‐2 KO, which is consistent with lower DGs (Figure 5C). We looked at the expression of the rate‐limiting enzyme in PG production, phosphatidylglycerophosphate synthase (PGS1). When compared to WT mice, PGS1 expression was elevated in 6‐ and 12‐month‐old but not in 14‐ to 16‐month‐old MKP‐2 KO mice (Figure 5D), although this difference was not statistically significant. We then looked at phosphatidylglycerophosphatase (PGPP), the enzyme that converts PGP into PG. When compared to WT mice, we observed a significant increase in PGPP in 6‐month‐old MKP‐2 KO mice, but not in 12‐ and 14–16‐month‐old MKP‐2 KO mice (Figure 5E). We observed decreased adiposity in 6‐, 12‐, and 14–16‐month‐old MKP‐2 KO as compared to WT controls (Figure 5F), which is consistent with decreased DGs. The results indicate a shift in adipose lipid metabolism characterized by increased HexCER and PG, alongside reduced DAGs. This pattern is associated with decreased DGAT1 expression and increased PGPP expression, suggesting altered DAG utilization and enhanced phosphatidylglycerol synthesis. These observations implicate MKP‐2 as an important regulator of lipid metabolism, and this has the potential to regulate adipose tissue development and biological aging.

### 3.6 Improved Metabolic Health and Lifespan Extension in Aging MKP‐2‐Deficient Mice

We looked at metabolic measures to determine how MKP‐2 affected biological aging after establishing its impact on adipose lipid composition, senescence, and SASPs in aged mice. We found that MKP‐2 deficiency improves metabolic health during aging. Compared with WT mice, middle‐aged and older male MKP‐2 KO mice had lower body weight, enhanced insulin sensitivity, and improved glucose tolerance (Figure 6A–H), extending previous observations in young MKP‐2 KO mice (Fernando et al. 2022). Next, we determined whether this improved metabolic health promotes longevity in MKP‐2 KO mice. Cohorts of male WT and MKP‐2 KO were monitored for maximum lifespan for more than three and a half years. Excitingly, we discovered that MKP‐2 KO mice increased median survival by 25% compared with WT mice (Figure 6I). These findings indicate that MKP‐2 deficiency promotes healthy aging by reducing adipose tissue senescence and improving metabolic health.

**FIGURE 6:**
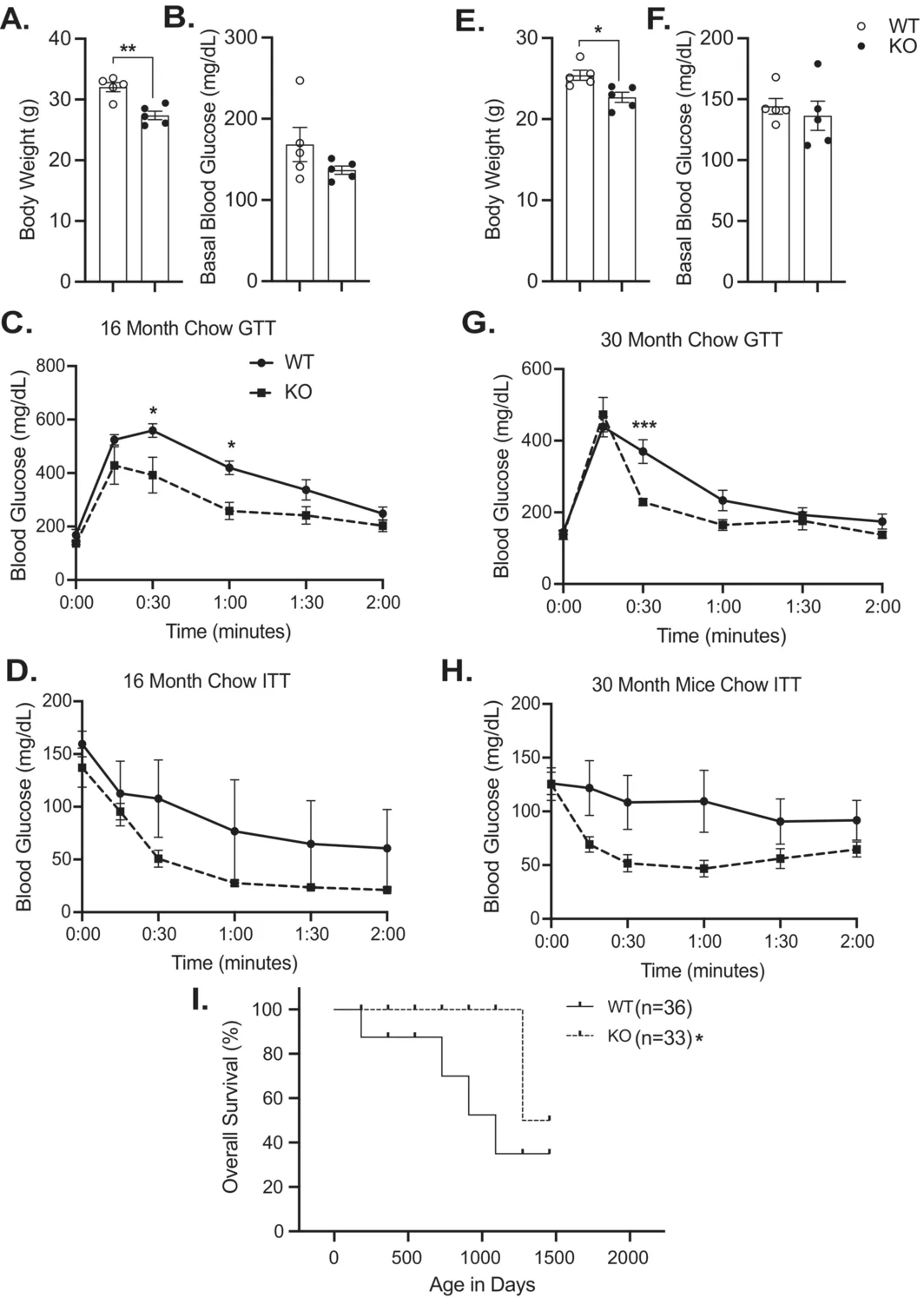
Glucose Homeostasis and Insulin Sensitivity in Aging MKP‐2 Deficient Mice. (A) Body weight, (B) Basal plasma glucose concentration, (C) Plasma glucose concentration during GTTs, (D) ITTs in 16‐month‐old chow‐fed male mice (E); body weight (F) basal plasma glucose concentration, (G) plasma glucose concentration during GTTs, and (H) ITTs in 30‐month‐old chow‐fed male MKP‐2 KO and WT mice (*n* = 5/genotype). (I) Kaplan–Meier overall survival curves of the denoted mice. Asterisks indicate significance to WT mice using logrank (Mantel‐Cox) tests. Results represent the mean ± SEM; *p < 0.05, **p < 0.01; ***p < 0.0001; as determined by *t*‐tests or analysis of variance (ANOVA) with Bonferroni's post‐test for multiple comparisons.

### 3.7 Enhanced MAPK and Akt Signaling in Adipose Tissue of MKP‐2‐Deficient Mice

The biology of adipose tissues has been shown to be significantly influenced by MAPK activity (Leiva et al. 2020; Nikolic et al. 2020), and it is crucial to test if MKP‐2 regulates this activity. We determined the phosphorylation status of ERK and JNK in EWAT. In 6–8‐month‐old chow‐fed MKP‐2 KO mice, we observed markedly increased JNK phosphorylation but not ERK phosphorylation as compared to WT mice (Figure 7A–C). Similarly, 16‐month‐old chow‐fed MKP‐2 KO mice exhibit significantly increased JNK phosphorylation but not ERK phosphorylation as compared to WT mice (Figure 7D–F). We next measured the phosphorylation status of Akt in EWAT of 16‐month‐old chow‐fed MKP‐2 KO mice. We found that in EWAT MKP‐2 KO mice, both Akt^Ser473^ and Akt^Thr308^ phosphorylation were significantly increased compared with WT mice (Figure 7G–I). These data demonstrate that MKP‐2 plays a role in negatively regulating JNK pathways in EWAT, and MKP‐2 deletion results in enhanced Akt activation that is associated with improved insulin sensitivity in MKP‐2 KO mice.

**FIGURE 7:**
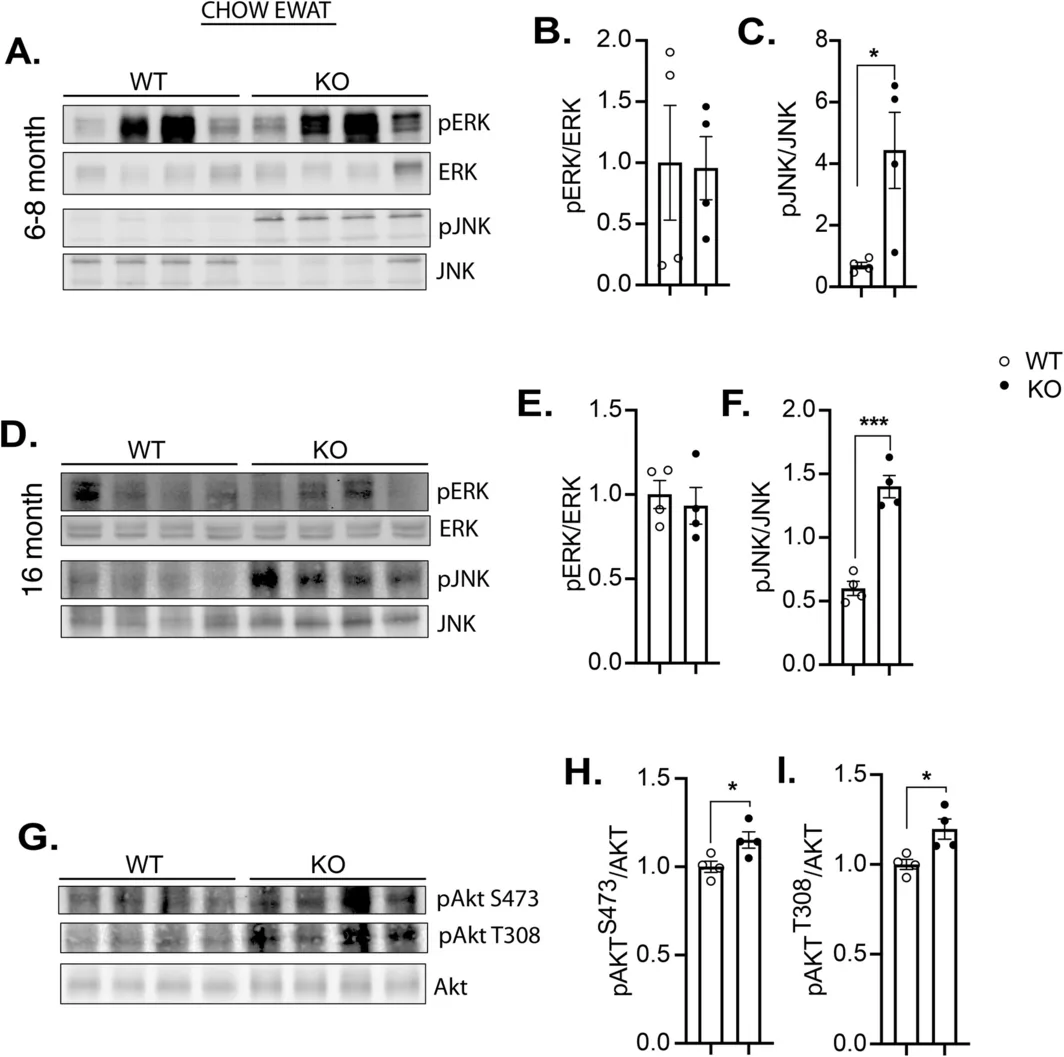
Enhanced MAPK Phosphorylation and Akt signaling in EWAT of MKP‐2‐deficient mice. EWAT tissue lysates from chow‐fed KO and WT mice (*n* = 3–4) were analyzed by immunoblotting. Representative immunoblots were quantitated by densitometry for phospho‐ERK/ERK, phospho‐JNK1/2/JNK1/2 (A–F), and phospho‐Akt/Akt (G–I). Results represent the mean ± SEM; *p < 0.05, ***p < 0.0001 as determined by student's *t*‐test.

## 4 Discussion

Understanding the molecular underpinnings by which MKP‐2 regulates cellular senescence and lipid metabolism could unveil novel molecular pathways for age‐related metabolic diseases. Using the MKP‐2 KO mouse model, we make important new observations regarding the functional role of MKP‐2 in vitro and in vivo. We identify adipose lipid remodeling as a potential component of the mechanism by which MKP‐2 deficiency promotes metabolic health and longevity. We identify a critical role for MKP‐2 in the regulation of premature senescence in MEFs and EWAT and lipid metabolism. We analyzed the metabolic characteristics of adipose tissue in male mice of different age groups and demonstrated that MKP‐2 deficiency leads to increased levels of specific glycosphingolipids, PGs, and DGs in adipose tissue. These lipid changes coincide with reduced pp53, p16, and PAI‐1, preservation of lamin B1, and increased phosphorylation of JNK and Akt in EWAT indicating that the relationship between adipose lipid metabolism, stress‐responsive signaling, cellular senescence, and aging may be significantly regulated by MKP‐2. We did not specifically evaluate whether the effects of MPK‐2 are sex‐dependent in the present study. Therefore, we cannot exclude the possibility that the response to MKP‐2 may differ between males and females. We acknowledge sex as a potential biological variable that should be addressed in future studies.

One interesting finding from our research is that MEFs derived from MKP‐2 KO mice exhibit resistance to premature senescence. We and others have previously shown that MKP‐2 is a critical regulator of cell survival and proliferation (Lawan et al. 2012; Ratsada et al. 2020). Maintaining long‐term senescence requires p16 expression (Serrano et al. 1997; Yamakoshi et al. 2009). According to a recent study, macrophages in particular that express high levels of p16 are characterized by many other senescence indicators, such as SA‐β‐gal, decreased proliferation, and associated SASP (Liu, Souroullas, et al. 2019). In vitro and in vivo, lamin B1 is a biomarker of senescence (Freund et al. 2012). Here, we demonstrate increased lamin B1 expression in MEFs and EWAT that are shielded from early senescence. Therefore, reduced p16 and elevated lamin B1 expression in MKP‐2‐deficient MEFs may contribute to the observed protection in senescence in MKP‐2 KO MEFs. Our data demonstrated upregulation of MKP‐2 in senescent MEFs compared with non‐senescent cells. This is consistent with a previous study that showed increased MKP‐2 protein levels in old senescent fibroblasts compared with young cells (Torres et al. 2003). Indeed, it is noteworthy that MEFs derived from MKP‐2‐deficient mice reciprocally phenocopy JNK null MEFs, since JNK mutant mice display senescence and increased p53 expression (Das et al. 2007). Another study showed that premature senescence results from a cascade of Bcl‐2 signaling that is triggered by loss of JNK activity (Lee et al. 2010). One well‐known indicator of both cellular senescence and organismal aging is telomere shortening (Liu, Wang, et al. 2019). We demonstrated that MKP‐2 KO MEFs displayed significantly longer telomere length compared to WT cells. This finding contributes to the body of research showing that MAPK phosphatases control cellular functions like cell cycle and proliferation and are now connected to telomere length regulation. PTEN, a protein tyrosine phosphatase, promotes telomere shortening (Seo et al. 2022). Consistent with this, we have previously demonstrated that the livers of MKP‐2‐deficient mice exhibited downregulated PTEN protein expression (Fernando et al. 2022). Together, these findings suggest that MKP‐2 is an important regulator of cellular senescence and that its absence may preserve cellular homeostasis and delay the establishment of the senescent phenotype, potentially through modulation of the JNK–p53–p16 axis.

The stress‐induced premature senescent phenotype of adipocytes may be attributed to the decreased expression and function of p53 and p16 in MKP‐2 KO EWAT. Obesity and metabolic syndrome are known to raise levels and activity of plasminogen activator inhibitor (PAI)‐1. Additionally, prior research has linked PAI‐1 to both insulin resistance and the growth of adipose tissue (Wang et al. 2018). Here, we demonstrate decreased PAI‐1 expression in EWAT, which is linked to decreased adiposity, senescence, and enhanced insulin sensitivity in MKP‐2 KO mice. Collectively, aging HFD‐fed male MKP‐2‐deficient animals exhibit decreased SA‐β‐gal activity, down‐regulation of p53, p16, lamin B1, and PAI‐1, and SASPs levels of IL‐6 and IL‐15. These findings suggest that MKP‐2 deficiency has a protective role in adipocyte senescence.

An important finding in this study is that MKP‐2‐deficient mice have a distinct lipidomic profile, which is mainly defined by a differential abundance of HexCERs, PGs, and DGs. Studies have shown that an increase in HexCER is metabolically beneficial (Reza et al. 2021). One study demonstrated that MEFs deficient in ceramide transfer protein (CERT) exhibit senescence and dysregulated sphingolipid metabolism by enhancing HexCERs secretion (Rao et al. 2007). Similarly, age‐related deficiencies in CD4(+) T cell activity and immunosenescence were caused by elevated HexCERs (Molano et al. 2012). In a long‐lived animal model, another study has shown that sphingolipid metabolism is disturbed (Lasher et al. 2024). Recent research suggests that lipid composition is closely linked to longevity, with long‐lived species showing genetic adaptations in lipid metabolism (Johnson and Stolzing 2019; Jobson et al. 2010; Fernàndez‐Bernal et al. 2025). Centenarians and their offspring have a unique lipid profile enriched in HexCERs (Fernàndez‐Bernal et al. 2025). We found evidence of higher abundance of HexCERs in the adipose tissue of MKP‐2 KO mice, suggesting this change in ceramide profiles is probably due to changes in ceramide metabolic pathways. Cers6 is highly expressed in adipose tissue and regulates C16‐Cer synthesis (Zeng et al. 2024). It has been observed that altering Cers6 expression improves insulin resistance and lowers C16:0 Cer levels (Raichur et al. 2019). Studies have shown a higher risk of diabetic nephropathy and the onset of cardiovascular disease in diabetic patients is negatively correlated with levels of lactosylceramide and HexCERs (Mandal et al. 2021; Düsing et al. 2023). The increase in HexCERs is of particular interest because sphingolipid metabolism has an important role in both metabolic regulation and cellular stress responses. Although ceramide accumulation is generally associated with insulin resistance and cellular dysfunction, conversion of ceramides into glycosylated derivatives can alter their biological activity, intracellular localization, and availability for downstream signaling (Jeffries and Krupenko 2018; Mitsutake et al. 2012; Kotronen et al. 2010). The increase in HexCERs in MKP‐2 KO adipose tissue may therefore indicate a shift in sphingolipid metabolism that limits the accumulation or signaling activity of more deleterious ceramide species (Molano et al. 2012; Apostolopoulou et al. 2018). Importantly, this change occurs in the context of reduced DGs and improved insulin signaling, suggesting that the overall lipidomic remodeling rather than any individual lipid class may contribute to the favorable metabolic phenotype. We found increased PG levels in MKP‐2 KO adipose tissue. Y.K. Cho et al. demonstrated that in human individuals, the amount of particular phospholipids, including PG, increases dramatically with age, which is consistent with the rise in different PG species in MKP‐2 KO adipose tissue (Cho et al. 2023). Furthermore, PG is a precursor molecule for the synthesis of cardiolipins; PG quantity is essential for mitochondrial biogenesis and function, indicating improved membrane lipid remodeling and increased antioxidant capacity in MKP‐2 KO mice adipose tissue, both of which are associated with healthy aging (Kojta et al. 2020). These findings could establish a causal connection between altered lipid metabolism observed in the MKP‐2‐deficient mice under conditions of obesity (Fernando et al. 2022). Future research on the mechanisms of MKP‐2 control of HexCERs, PGs and DGs in senescence in adipose tissue may build on this work.

Previous observations have established that MKP‐2 KO mice exhibit a metabolically favorable phenotype characterized by reduced food intake, adiposity, respiratory exchange ratio (RER), and hepatic triglyceride (TG) accumulation (Fernando et al. 2022). The present findings provide additional insight into the potential mechanisms underlying these phenotypes by demonstrating substantial remodeling of the adipose lipidome in MKP‐2‐deficient mice. Specifically, EWAT from MKP‐2 KO mice displayed increased HexCERs and PG, together with reduced DGs. These lipidomic changes were accompanied by reduced markers of cellular senescence, enhanced insulin signaling, and increased longevity. Collectively, the findings suggest that MKP‐2 deficiency promotes an adipose metabolic state characterized by altered lipid partitioning, reduced cellular stress, and preservation of insulin responsiveness, which may contribute to the systemic metabolic and aging benefits observed in these animals.

An important feature of this metabolic remodeling is its association with reduced adipose cellular senescence. EWAT from MKP‐2 KO mice exhibited reduced phosphorylated p53, p16, and PAI‐1, alongside increased lamin B1. The coordinated changes in these markers are consistent with attenuation of the senescence phenotype. Particularly, preservation of lamin B1, which is frequently lost during cellular senescence (Freund et al. 2012), together with reduced p16 and PAI‐1, supports the interpretation that MKP‐2 deficiency is associated with reduced accumulation or activation of senescent cells in adipose tissue. Furthermore, the p53 and p16 pathways play a key role in cellular senescence. Research indicates that p53 controls lipid metabolism and that JNK inhibition stimulates p53‐dependent senescence, suggesting possible connections between MAPK signaling, aging, and lipid regulation (Lawan et al. 2012; Zeng et al. 2024; Hamsanathan and Gurkar 2022). We are unable to conclude with certainty whether adipocytes, stromal cells, or both cell groups are the primary sites of senescence based on our available data. Since the senescence‐related alterations seen in our studies were evaluated at the tissue level, it is impossible to definitively link them to a particular cell type. To identify which populations experience senescence, future research utilizing cell‐type‐specific identification in conjunction with senescence markers will be necessary.

The relationship between altered adipose lipid metabolism and senescence is biologically plausible. Chronic lipid excess and accumulation of bioactive lipid intermediates can generate oxidative, inflammatory, and endoplasmic reticulum stress, all of which can promote cellular senescence (Regulski 2017; Calcinotto et al. 2019). Conversely, reducing adipose lipid stress may attenuate activation of p53‐ and p16‐dependent pathways. The reduced DG content and altered sphingolipid composition observed in MKP‐2 KO mice may therefore contribute to the suppression of senescence‐associated signaling. In this model, the favorable lipid environment of MKP‐2‐deficient adipose tissue could reduce chronic cellular stress, preserve nuclear integrity as reflected by lamin B1, and suppress downstream senescence‐associated factors such as PAI‐1. We acknowledge that the use of aged, HFD‐fed mice for senescence‐related experiments and younger, chow‐fed mice for lipidomic analyses represents an important experimental limitation. Because these experiments were conducted in animals differing in both age and dietary conditions, the observed lipid remodeling cannot be directly attributed to senescence, independent of age‐ or diet‐related effects. Consequently, the relationship between cellular senescence and lipid remodeling remains associative rather than directly established, and future studies using age‐ and diet‐matched experimental groups are needed to clarify this relationship.

The changes in JNK and Akt phosphorylation provide an additional molecular link between the lipidomic phenotype and improved metabolic function. Increased JNK phosphorylation in EWAT of MKP‐2 KO mice, despite the reduction in senescence markers, suggests that MKP‐2 deficiency does not simply suppress all stress‐responsive MAPK signaling. Rather, the increase in JNK phosphorylation may reflect an adaptive signaling response associated with altered nutrient utilization or adipose remodeling. Importantly, this increase occurs together with enhanced Akt phosphorylation, indicating that JNK activation in this setting is not accompanied by impaired insulin signaling. The increased Akt phosphorylation is consistent with improved insulin responsiveness and may represent a key molecular correlate of the reduced DG abundance and improved systemic glucose metabolism. This finding is particularly noteworthy because JNK is commonly associated with metabolic stress and insulin resistance (Lawan et al. 2015; Westenberger et al. 2021). The present results therefore suggest that the functional consequences of JNK phosphorylation may be context dependent and that MKP‐2 deficiency may alter the balance between JNK and insulin‐signaling pathways rather than simply suppressing JNK activity. Enhanced Akt phosphorylation despite increased JNK phosphorylation could indicate that the adipose tissue of MKP‐2 KO mice retains insulin responsiveness in the presence of altered MAPK signaling. Alternatively, the increased JNK phosphorylation may represent a compensatory or adaptive response that is uncoupled from the inhibitory effects typically associated with chronic metabolic stress. Further assessment of JNK substrates, insulin receptor signaling intermediates, and temporal responses to insulin will be important for distinguishing these possibilities.

It is especially compelling that these alterations are linked to longer lifespan. Metabolic dysfunction, ectopic lipid accumulation, impaired insulin signaling, and cellular senescence are all recognized contributors to aging and age‐related disease (He and Sharpless 2017; Johnson and Stolzing 2019). The MKP‐2 KO phenotype combines attenuation of several of these processes, including reduced adiposity, improved insulin signaling, favorable adipose lipid remodeling, and reduced senescence‐associated signaling. It is therefore reasonable that the adipose metabolic state established by MKP‐2 deficiency contributes to a systemic environment that promotes healthy aging and lifespan extension. Reduced adipose senescence may be especially important because senescent adipose cells can produce inflammatory and matrix‐remodeling factors that adversely affect systemic metabolism (Frasca and Blomberg 2020). Suppression of this phenotype could consequently reinforce the improvements in insulin sensitivity and lipid metabolism. The current findings demonstrate strong associations but do not establish a causal relationship between individual lipid classes and the anti‐senescent or longevity phenotypes. Whether increased HexCERs or PG directly promote metabolic health, whether reduced DGs are sufficient to improve insulin signaling, and whether these lipid changes are downstream of altered MKP‐2/JNK signaling remain to be determined. Adipose‐specific MKP‐2 deletion, pair‐feeding experiments, lipid flux measurements, and targeted manipulation of sphingolipid, DG, and PG metabolism will be useful for distinguishing direct effects of MKP‐2 from consequences of reduced caloric intake.

## 5 Conclusions

Aging is a complex biological process characterized by a gradual decline in physiological functions, impaired cellular repair mechanisms, and increased disease susceptibility (Von Bank et al. 2021; van den Berg 2020). Understanding the fundamental cellular pathways and mechanisms that regulate aging can expand our knowledge of healthy aging and lifespan extension. In summary, these findings suggest that adipose lipid remodeling is associated with the metabolic health and longevity phenotypes observed in MKP‐2‐deficient mice. MKP‐2 KO mice exhibited increased adipose HexCERs and PG and decreased DGs. These lipid changes coincide with reduced pp53, p16, and PAI‐1, preservation of lamin B1, and increased phosphorylation of JNK and Akt in EWAT. The resulting phenotype is characterized by reduced cellular senescence and enhanced insulin responsiveness and is associated with extended longevity. Thus, MKP‐2 may function in the regulation of adipose lipid metabolism, stress‐responsive signaling, cellular senescence, and aging. The observed changes associated with MKP‐2 deficiency suggest a potential relationship between MKP‐2 and adipose metabolic adaptations linked to systemic health and lifespan, although the causal relationships remain to be established.

## Author Contributions

Ahmed Lawan conceived, designed and wrote the manuscript. Morgan Welch, Nabin Ghimire, Shauri Smith, Cassandra Secunda, Alexis Fink, Alissar Sleiman, Laura Chambliss, Christopher Pinckard, Chloe Reyes, Priya Katta, Anna Ai performed experiments and analyzed data. Benjamin R. Harrison analyzed the lipidomics data. As the work's guarantor, Ahmed Lawan has complete access to all study data and is accountable for the data's accuracy and integrity.

## Funding

This work was supported by The University of Alabama in Huntsville (251539), San Antonio Nathan Shock Center Pathology Core (P30AG13319), and University of Washington Nathan Shock Center (P30AG013280).

## Conflicts of Interest

The authors declare no conflicts of interest.

## Supporting information


**Table S1:** Structural features of the adipose tissue lipidome.

## Data Availability

Upon reasonable request, the corresponding author will provide the data supporting the study's conclusions.
