# Supplementary material for: MKP‐2 Genetic Depletion Modulates Adipose Lipid Composition, Cellular Senescence, and Aging in Mice
Source: Aging Cell. 2026 Nov 3;25(11):e70794. doi: 10.1111/acel.70794 (PMC13732110; doi:10.1111/acel.70794)
Supplement: Supplementary file 1 [file ACEL-25-e70794-s001.docx]

**MKP-2 genetic depletion modulates adipose lipid composition, cellular senescence, and aging in mice**

Morgan Welch^1,2^, Nabin Ghimire^1^, Shauri Smith^1,4^, Cassandra Secunda^1,3^, Alexis Fink^1^, Alissar Sleiman^1^, Laura Chambliss^1^, Christopher Pinckard^1^, Chloe Reyes^1^, Priya Katta^1^, Anna Ai^1^, Benjamin R Harrison^5^, and Ahmed Lawan^1*^

*^1^Department of Biological Sciences, University of Alabama in Huntsville, Huntsville, Alabama 35899, USA*

*^5^Department of Anesthesiology and Pain Medicine, University of Washington, Seattle, WA 98195, USA*

*#2 current address: Aviagen North America, Elkmont, AL 35620, USA*

*#3 current address: Department of Microbiology and Microbial Genetics, University of Pittsburgh School of Medicine, Pittsburgh, PA 15219, USA*

*#4 current address: PingWind Inc, Annandale, VA 22003, USA*

**Keywords:** Senescence, aging, MKP-2, MAPK, adipose tissue

**Running Title:** Loss of MKP-2 alters senescence and lipid composition in aged adipose tissue.

*To whom correspondence should be addressed.

Ahmed Lawan, Ph.D.,

University of Alabama in Huntsville,

Department of Biological Sciences

SST 369H, 301 Sparkman Drive,

Huntsville, AL 35899, United States,

Tel: 256-824-6264

Email: al0122@uah.edu

**Table 1.** Structural features of the adipose tissue lipidome

| **Species** | **Lipid class** | **Fatty acid 1** | **Fatty acid 2** |
| --- | --- | --- | --- |
| DG 14:0_18:1 | Diacylglycerol | 14:0 | 18:1 |
| DG 14:0_18:2 | Diacylglycerol | 14:0 | 18:2 |
| DG 15:0_18:1 | Diacylglycerol | 15:0 | 18:1 |
| DG 16:0_16:1 | Diacylglycerol | 16:0 | 16:1 |
| DG 16:0_18:0 | Diacylglycerol | 16:0 | 18:0 |
| DG 16:0_18:1 | Diacylglycerol | 16:0 | 18:1 |
| DG 16:0_18:2 | Diacylglycerol | 16:0 | 18:2 |
| DG 16:0_18:3 | Diacylglycerol | 16:0 | 18:3 |
| DG 16:0_20:3 | Diacylglycerol | 16:0 | 20:3 |
| DG 16:1_16:1 | Diacylglycerol | 16:1 | 16:1 |
| DG 16:1_18:0 | Diacylglycerol | 16:1 | 18:0 |
| DG 16:1_18:1 | Diacylglycerol | 16:1 | 18:1 |
| DG 18:0_18:1 | Diacylglycerol | 18:0 | 18:1 |
| DG 18:0_18:2 | Diacylglycerol | 18:0 | 18:2 |
| DG 18:1_18:1 | Diacylglycerol | 18:1 | 18:1 |
| DG 18:1_18:2 | Diacylglycerol | 18:1 | 18:2 |
| DG 18:1_20:0 | Diacylglycerol | 18:1 | 20:0 |
| DG 18:1_20:1 | Diacylglycerol | 18:1 | 20:1 |
| DG 18:2_18:3 | Diacylglycerol | 18:2 | 18:3 |
| HexCER d18:1_22:0 | Hexosylceramides | d18:1 | 22:0 |
| HexCER d18:1_22:1 | Hexosylceramides | d18:1 | 22:1 |
| HexCER d18:1_24:0 | Hexosylceramides | d18:1 | 24:0 |
| PG 14:0_17:0 | Phosphatidylglycerol | 14:0 | 17:0 |
| PG 14:0_18:1 | Phosphatidylglycerol | 14:0 | 18:1 |
| PG 14:0_18:2 | Phosphatidylglycerol | 14:0 | 18:2 |
| PG 16:0_16:1 | Phosphatidylglycerol | 16:0 | 16:1 |
| PG 16:0_17:0 | Phosphatidylglycerol | 16:0 | 17:0 |
| PG 16:0_18:0 | Phosphatidylglycerol | 16:0 | 18:0 |
| PG 16:0_18:1 | Phosphatidylglycerol | 16:0 | 18:1 |
| PG 16:0_18:2 | Phosphatidylglycerol | 16:0 | 18:2 |
| PG 16:0_20:3 | Phosphatidylglycerol | 16:0 | 20:3 |
| PG 16:0_20:4 | Phosphatidylglycerol | 16:0 | 20:4 |
| PG 16:1_18:1 | Phosphatidylglycerol | 16:1 | 18:1 |
| PG 16:1_18:2 | Phosphatidylglycerol | 16:1 | 18:2 |
| PG 17:0_18:0 | Phosphatidylglycerol | 17:0 | 18:0 |
| PG 17:0_18:1 | Phosphatidylglycerol | 17:0 | 18:1 |
| PG 17:0_18:2 | Phosphatidylglycerol | 17:0 | 18:2 |
| PG 17:0_20:0 | Phosphatidylglycerol | 17:0 | 20:0 |
| PG 17:0_20:1 | Phosphatidylglycerol | 17:0 | 20:1 |
| PG 17:0_20:3 | Phosphatidylglycerol | 17:0 | 20:3 |
| PG 17:0_22:1 | Phosphatidylglycerol | 17:0 | 22:1 |
| PG 17:0_22:2 | Phosphatidylglycerol | 17:0 | 22:2 |
| PG 18:0_18:0 | Phosphatidylglycerol | 18:0 | 18:0 |
| PG 18:0_18:1 | Phosphatidylglycerol | 18:0 | 18:1 |
| PG 18:0_18:2 | Phosphatidylglycerol | 18:0 | 18:2 |
| PG 18:0_20:4 | Phosphatidylglycerol | 18:0 | 20:4 |
| PG 18:1_18:1 | Phosphatidylglycerol | 18:1 | 18:1 |
| PG 18:1_18:2 | Phosphatidylglycerol | 18:1 | 18:2 |
| PG 18:1_20:0 | Phosphatidylglycerol | 18:1 | 20:0 |
| PG 18:1_20:3 | Phosphatidylglycerol | 18:1 | 20:3 |
| PG 18:1_20:4 | Phosphatidylglycerol | 18:1 | 20:4 |
| PG 18:1_22:6 | Phosphatidylglycerol | 18:1 | 22:6 |
| PG 18:2_18:2 | Phosphatidylglycerol | 18:2 | 18:2 |
| PG 18:2_20:0 | Phosphatidylglycerol | 18:2 | 20:0 |
| PG 18:2_20:3 | Phosphatidylglycerol | 18:2 | 20:3 |
| PG 18:2_20:4 | Phosphatidylglycerol | 18:2 | 20:4 |
| PG 20:0_20:4 | Phosphatidylglycerol | 20:0 | 20:4 |
